# Patient perception of recovery following severe trauma: experience from a trauma follow-up clinic

**DOI:** 10.1186/1757-7241-22-S1-P11

**Published:** 2014-07-07

**Authors:** Anita West, Karen Hoffman

**Affiliations:** 1 Barts Health NHS Trust, Royal London Hospital, Trauma Unit, Whitechapel, London, E1 1BB, UK; 2 Queen Mary University London, Blizard Institute, 4 Newark Street, London E1 2AT, UK

## Background

Appropriate follow-up for survivors of trauma and their family is important. However, many clinician led follow-up clinics focus on single systems of injury rather than the overall recovery of the patient. This study examined patient perception of outcome following multiple injuries at a Major Trauma Centre.

## Methods

A prospective cohort study was conducted on trauma patients admitted to a Major Trauma Centre for more than 72 hours. A proportion of these patients were seen in a trauma follow-up clinic approximately two months post injury. Semi structured interviews were conducted during clinic appointments. The World Health Organisation International Classification of Function Disability and Health (WHO ICF) was used as a framework to structure questions relating to recovery. Descriptive data analysis was completed.

## Results

Two hundred and ninety seven patients were recruited with a median ISS of 13. The median age was 29 years and 57% of patients suffered penetrating trauma. There was a difference in the type of problems reported between blunt and penetrating trauma patients. Seventy percent of patients reported pain and mental health as limitations to recovery. Additional participation restrictions, such as environmental barriers, fatigue and return to work were highlighted as problematic.

## Conclusions

The ICF was a useful framework to capture a vast range of health components during a follow-up clinic attendance. It was able to describe the differences in patient populations. Interviews emphasised the complex psycho-social needs of trauma patients in an urban setting.

